# Structural insights into the unique inhibitory mechanism of the silkworm protease inhibitor serpin18

**DOI:** 10.1038/srep11863

**Published:** 2015-07-07

**Authors:** Peng-Chao Guo, Zhaoming Dong, Ping Zhao, Yan Zhang, Huawei He, Xiang Tan, Weiwei Zhang, Qingyou Xia

**Affiliations:** 1 State Key Laboratory of Silkworm Genome Biology, Southwest University, 216, Tiansheng Road, Beibei, Chongqing 400716, People's Republic of China

## Abstract

Serpins generally serve as inhibitors that utilize a mobile reactive center loop (RCL) as bait to trap protease targets. Here, we present the crystal structure of serpin18 from *Bombyx mori* at 1.65 Å resolution, which has a very short and stable RCL. Activity analysis showed that the inhibitory target of serpin18 is a cysteine protease rather than a serine protease. Notably, this inhibitiory reaction results from the formation of an intermediate complex, which then follows for the digestion of protease and inhibitor into small fragments. This activity differs from previously reported modes of inhibition for serpins. Our findings have thus provided novel structural insights into the unique inhibitory mechanism of serpin18. Furthermore, one physiological target of serpin18, fibroinase, was identified, which enables us to better define the potential role for serpin18 in regulating fibroinase activity during *B. mori* development.

Serpins play important roles in multiple processes, such as blood coagulation, fibrinolysis, tumor metastasis and immunity^1^,^2^,^3^. Mature serpins usually contain 350–400 amino acids and share a conserved folding pattern that is mainly composed of eight or nine α-helices, three β-sheets, and a reactive center loop (RCL). The exposed RCL is located near the carboxyl-terminus, and it acts as bait for protease binding and cleavage. The name ‘serpin’ was originally derived from the fact that most serpins were identified as serine protease inhibitors^4^. However, recent studies have shown that several serpins exhibit inhibitory activity against cysteine proteases. For example, viral serpin crmA^5^ and human serpin PI9^6^ can inhibit members of the caspase family, and serpin squamous cell carcinoma antigen 1^7^,^8^ can inhibit cathepsins K, L, and S. Additionally, the plasma serpin inhibitor of coagulation protease, antithrombin, has been shown to inactivate papain^9^ and Arg-gingipain (bacterial cysteine protease)^10^. Furthermore, some members of the superfamily lack any protease inhibitory property, including ovalbumin^11^, angiotensinogen^12^, and thyroxine binding globulin^13^,^14^.

Inhibition of serine protease occurs when the mobile RCL of the serpin forms a covalent complex with the target serine protease, thereby blocking the activity of the protease. Meanwhile, the formation of a serpin-protease complex requires that a portion of the RCL inserts into β-sheet A of the serpin protein, thereby carrying the covalently bound protease from the top to the bottom of the serpin. The inhibition reaction can generate two reaction products, the covalent 1:1 serpin-protease complex, or the RCL-cleaved serpin (stably inactive serpin)^2^,^15^. The mechanism of cysteine protease inhibition is similar except no stable serpin-cysteine protease complex is observed. Instead, the RCL-cleaved serpin is the most dominant product. After these inhibitory reactions, the protease moiety has much high susceptibility to proteolysis^16^, whereas the hydrolysis of the inhibitor is very slow^17^.

Serpins are widely distributed in eukaryotes and some viruses that infect them, and are even found in some prokaryotes^18^. Three homologous serpins have been identified in the silk gland (a highly specialized organ that functions to synthesize and store silk proteins) of *Bombyx mori*: serpin16, serpin18, and serpin22^19^,^20^,^21^. These three serpins are mainly distributed in the middle silk gland and contain a signal peptide for secretion^19^. They also share high sequence homology (~87%), implying that they might carry out a similar and specific function in the middle silk gland lumen. To understand the physiological roles and inhibitory mechanism of these serpins, we determined the crystal structure of serpin18 at 1.65 Å. This structure enabled us to identify a novel RCL of serpins. Structural and biochemical analysis revealed that serpin18 inactivates the cysteine protease through a unique inhibitory mode involving the digestion of protease and inhibitor. Furthermore, we identified one physiological target of serpin18, and proposed its biological role in regulating protease activity during the development of *B. mori*.

## Results

### Overall structure

**Figure 1:**
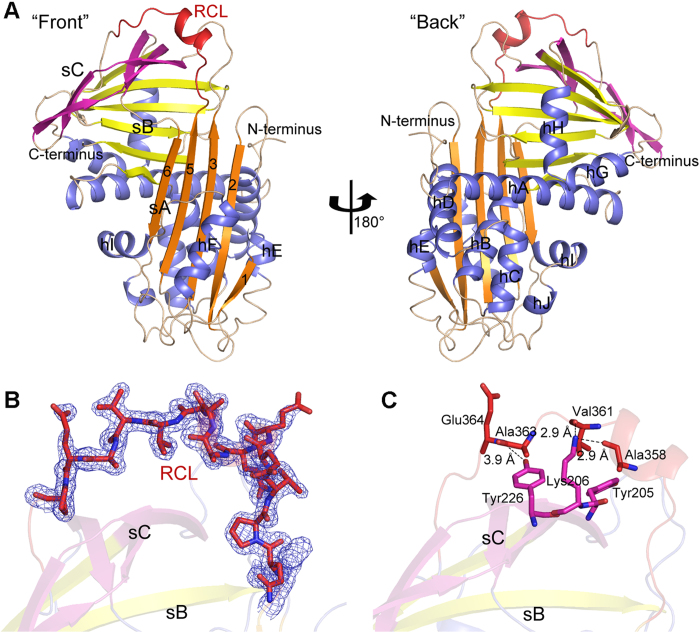
Overall structure of serpin18. (**A**) Schematic representation of serpin18 fold. Blue, helical region; red, RCL; and the three β-sheets are distinguished by diffferent colour (sA, orange; sB, yellow; sC, magenta). Secondary elements are labeled, and the specific numbering of strands 1-3, 5, and 6 of sA is shown. (**B**) The residues in RCL are emphasized in stick presentation with the corresponding electron density at a sigma level of 1.0 in the *2Fo-Fc* map. (**C**) Residues involved the interactions between the center of RCL and sC are shown with sticks, and the hydrogen bonds are black dashed lines. All figures were prepared using *PyMOL*.

The overall fold of serpin18 resembles the canonical serpin conformation: an α/β fold consisting of one core β-sheets surrounded by nine α-helices (hA–hI) that are classified according to the conventional serpin nomenclature^22^. The core β-sheets includes three antiparallel β-sheets (sA: β1, β2, β3, β5, and β6; sB; and sC) (Fig. 1A and Fig. S1). Additionally, one RCL, which functions as the bait for the target protease in classical serpins, is solvent-exposed and located on the top of the molecule. Specifically, the segment with residues Glu352–Pro368 makes up the active RCL region, which is folded into a 1.5-turn helix (Glu355–Ser360) and two defined loops (Gln352–Thr354 and Gln361–Pro365, respectively). All residues of the RCL were well defined in the final electron density map (Fig. 1B), presumably because of the interaction between the RCL region and the underlying protein scaffold. Notably, the carbonyl oxygens of Val361 and Ala358 make two hydrogen bonds with Nζ of Lys206. The amide nitrogen of Glu364 forms a hydrogen bond with the hydroxyl group of Tyr226. Moreover, hydrophobic contacts between the aliphatic side chains of Val361 and Tyr205, with Ala361 and Tyr226, respectively, also contribute to the stable conformation of RCL (Fig. 1C).

### Structural comparisons to other serpins

**Figure 2:**
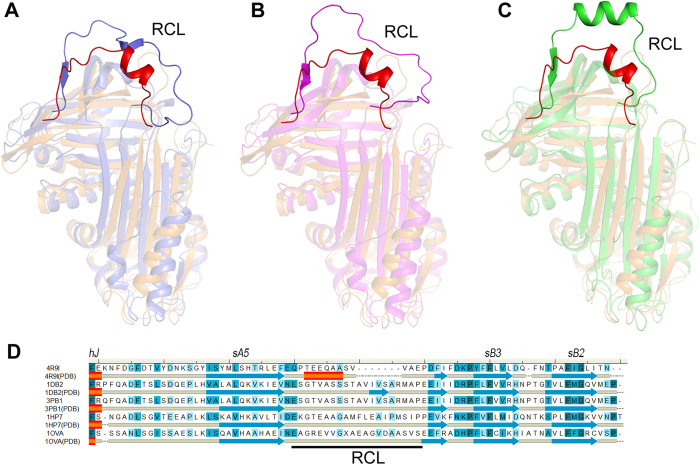
Close-up view of RCL. (**A**) Comparision of the overallstructure between serpin18 (orange) and human plasminogen activator inhibitor-1 (PDB: 1DB2, blue), (**B**) human plasminogen activator inhibitor-1 complexed with plasminogen activator (PDB: 3PB1, lightmagenta) and (**C**) Ovalbumin (PDB: 1OVA, green). All figures were prepared using *PyMOL*. The reactive center loop of serpin18 and the counterparts of serpins are highlight. (**D**) Structure-based multialignment of *Bombyx mori* serpin18 (NP_001139711.1) and 1DB2, *Homo sapiens* plasminogen activator inhibitor-1 (NP_000593.1; Identity, 18.8%; RMSD, 4.2 Å); 3PB1, *Homo sapiens* plasminogen activator inhibitor-1 ((NP_000593.1; Identity, 18.6%; RMSD, 4.6 Å ); 1HP7, *Homo sapiens* alpha-1-antitrypsin (NP_000286.3; Identity, 21.3%; RMSD, 4.5 Å) and 1OVA, *Gallus gallus* ovalbumin (NP_001073231.1; Identity, 16.9%; RMSD, 4.3 Å). The second structure elements are indicated under the primary sequences, and the conserved residues are shaded in blue. The multialignment was generated based on the structural superposition^44^.

To determine the differences between serpin18 and the other classical serpins, we superimposed serpin18 against another three structures of uncleaved serpins that had been previously deposited in the Protein Data Bank. Superposition of serpin18 against all counterparts yielded an overall root mean square deviation (RMSD) in the range of 4.2–4.6 Å over ~360 Cα atoms, implying that they adopt a distinct conformation despite sharing a similar structure topology (Fig. 2A–C). In a careful analysis of the helical region, we found that the helical region of serpin18 adopts a pattern similar to other serpins. Within the core domain, the three β-sheets in serpin18 are bent more closely towards the core of the protein compared to those of other serpins, especially β-sheet C. Notably, the counterpart of this short helix in typical serpin molecules is relaxed into a β-strand (designed sC1) in serpin18 and is bent towards the core of the molecule, leaving space for the short helix of RCL (Fig. S2).

Nevertheless, the major differences between serpin18 and these counterparts are in the RCL regions. The RCL in serpin18 is situated directly above of β-sheet C, and is closer to the core of the protein compared to those of the classical serpins. Further structural analysis showed that the RCL in serpin18 adopts a more stable conformation (Fig. 1B), presumably because of interactions between the RCL center region and β-sheet C. Furthermore, multiple-sequence alignment and structural analyses showed that the RCL in serpin18 is shorter by about 7 amino acids compared to that of the typical serpins. The RCL of typical serpins is composed of about 20 amino acids (from P17 to P3′), whereas the RCL of serpin18 has only a total of 13 amino acids (Fig. 2D). These differences in the RCL between serpin18 and its counterparts imply that serpin18 might possess a distinct target specificity^23^.

### The inhibitory activity and reactive site of serpin18

**Figure 3:**
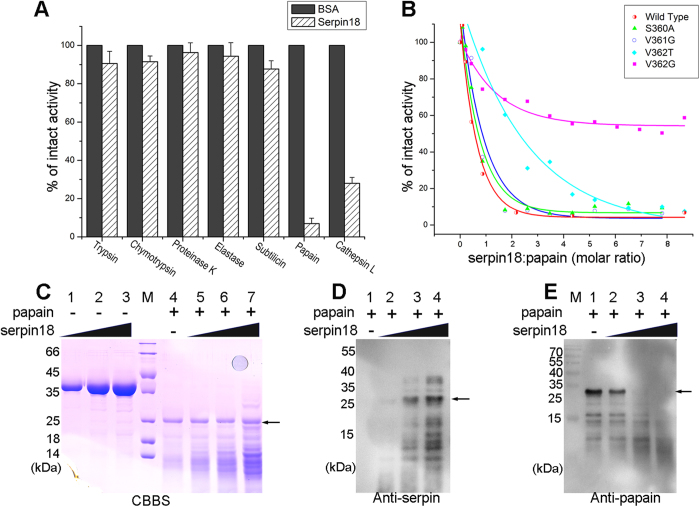
The inhibitory activity of serpin18. (**A**) The bar graph diagrams of residual activities of proteases incubated with serpin18 at molar ratios (inhibitor: protease) of 5. (**B**) Titration of papain with the wild-type serpin18 and variants, monitored by the loss of papain activity. (**C**) SDS-PAGE of the reaction between serpin18 and papain were analysed by Coomassie brilliant blue staining (CBBS). Lanes 1 to 3: 0.5 mg/ml, 1.0 mg/ml, 1.5 mg/ml serpin18 without papain; M, protein marker; Lane 4, 0.5 mg/ml papain; Lane 5–7, samples corresponding to lanes 1 to 3, respectively, with 0.5 mg/ml papain. Western blot analysis of the reaction between serpin18 and papain with antibodies against (**D**) anti-serpin and (**E**) anti-papain under the denaturing conditions. Lane 1, 0.5 mg/ml papain; Lanes 2 to 4: 0.5 mg/ml, 1.0 mg/ml, 1.5 mg/ml serpin18 with 0.5 mg/ml papain. The gels were run under the same experimental conditions.

To evaluate the inhibitory activity of serpin18, the candidate proteases trypsin, chymotrypsin, elastase, subtilisin, protease K, and papain were each incubated with the inhibitor. The residual proteolytic activity of trypsin, chymotrypsin, elastase, subtilisin, and protease K were each reduced by 4%–10%, implying that serpin18 has almost no inhibitory activity towards these serine proteases (Fig. 3A). Interestingly, serpin18 inhibited the proteolytic activity of papain (a cysteine protease) by 90% in a dose-dependent fashion (Fig. 3A,B). Moreover, the papain-like mammalian cysteine protease, cathepsin L, was also inactivated by serpin18 (Fig. 3A). These results demonstrated that serpin18 might act as a cysteine protease inhibitor, instead of as a serine protease inhibitor.

In the serpin18 structure, three solvent exposed residues located at the top of the molecule (Ser360, Val361, and Val362) are candidate residues at the reactive site. To confirm this crystallographic observation, we performed a series of site-directed mutagenesis experiments and tested the inhibitory activities of the mutants. The inhibitory activities of S360A and V361G showed little change compared to that of wild-type (Fig. 3B). In contrast, the inhibitory activities of the V362G and V362T mutants dramatically decreased, resulting in an extrapolated molar ratio of inhibitor-to-protease, which increased from ~1.3 for the wild-type to ~5–7 for the Val362 variants. This result confirmed the importance of Val362 at the reactive site of serpin18. Notably, the length of the RCL up to the scissile bond in serpin18 is only 11 amino acids, whereas the length in other serpins is always 17 residues (P17–P1)^23^.

### Analyses of serpin-papain reactions by PAGE

To elucidate how serpin18 inhibits papain/cysteine protease, the reaction mixture of serpin18 and papain was semi-quantatively analyzed using SDS–PAGE and Native-PAGE (Fig. 3C–E, and S3). The apparent molecular weight of the serpin18-papain complex was predicted to be ~66 kDa, whereas no corresponding band was detected by either Coomassie staining (Fig. 3C) or western blot analysis (Fig. 3D,E) under the denaturing conditions. However, the intensity of the serpin18 band was greatly reduced, and a peptide band with an apparent molecular weight of 26 kDa became stronger with increased of serpin18 levels (Fig. 3C, lane 5–7). Western blot analysis demonstrated that the band was mainly composed of the digested serpin18 fragments (Fig. 3D), implying that the inactivation of papain is accompanied by the pronounced cleavage of serpin18. This is a unique phenomenon that differs from any reported inhibition of serine proteases and/or cysteine proteases with serpins^23^. For papain, western blot analysis showed that the papain band progressively disappeared and several shorter fragments appeared with increasing amounts of serpin18 (Fig. 3E). These findings indicated that papain had been digested into smaller fragments during the incubation with serpin18, and this phenomenon could also be observed for interactions between human α-antithrombin and papain^24^. In addition, Native-PAGE analysis of the reaction mixtures of serpin18 and papain showed that no band behaving as a complex was appeared under the non-denaturing conditions (Fig. S3). Accordingly, the reduction in papain activity probably results from the formation of an intermediate complex with serpin, rather than a stable serpin-cysteine protease complex, and subsequently involves the degradation of papain and serpin into small fragments.

### The expression and localization of serpin 18 and its paralogs

**Figure 4:**
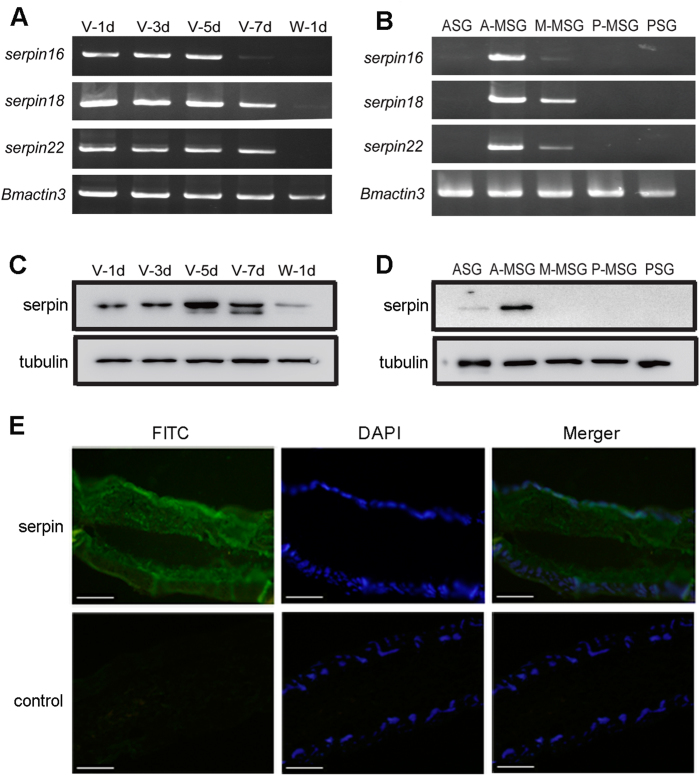
Expression patterns and localization of serpins in the silk gland. (**A**) Expression patterns of *serpin16, serpin18 and serpin22* genes in the day1, 3, 5, 7 of the fifth instar (V-1d, V-3d, V-5d, V-7d), and the hour 12 after wandering (W-1d). (**B**) Expression patterns of serpin were from female or male larvae in the different segments of the silk gland in the day 5 of the fifth instar. Western blot analysis for serpin18 and its paralogs in the (**C**) developmental stages of the fifth instar and (**D**) different segments of the silk gland. The silkworm tubulin was used as internal control. The gels were run under the same experimental conditions and the full length blots and gels are presented in Supplementary Figure S6. (**E**) Immunofluorescence analysis of serpin18 and its paralogs in the A-MSG on fifth day of the fifth instar. Slides were incubated with anti-serpin16 antibody followed by the secondary antibody labeled with FITC (greeen) and counterstained with DAPI (blue). Control experiments were also performed using pre-immune serum. Bar, 100 μm.

To elucidate the physiological roles of serpins in the silk gland, semi-quantitative RT–PCR was used to analyze the temporal–spatial expression profiles of *serpin18* and its paralogs (*serpin16* and *serpin22*). These *serpins* showed similar expression patterns during the silkworm development: expression levels increased from the first to the fifth day, decreased on the seventh day, and disappeared by the wandering stage (Fig. 4A). We then further divided the silk gland into five morphologically and functionally distinct compartments (anterior silk gland, ASG; anterior/middle/posterior regions of the middle silk gland, A/M/P-MSG; and posterior silk gland, PSG; Fig. S4), and then investigated the expression patterns in each region on the fifth day of the fifth instar. We found that all three serpins were expressed exclusively in the MSG, with the high expression levels in the A-MSG and the low levels in the M-MSG (Fig. 4B).

To explore the expression pattern of all the three serpin proteins, we performed western blot analysis using a polyclonal antibody against serpin16, an antibody that exhibits cross reactivity with all three serpins as a consequence of sequence similarities (see Supplementary Information). The temporal expression of the three serpins showed dynamic changes from the first day of the fifth instar to the wandering stage (Fig. 4C): the expression pattern showed a parabola shape with a peak on the fifth or seventh day, then abruptly declined and ultimately disappeared by the wandering stage. The spatial expression profile showed that serpins were mainly distributed in the A-MSG on the fifth day of the fifth instar larvae (Fig. 4D), which is consistent with the semi-quantitative RT–PCR results (Fig. 4B). Furthermore, immunofluorescence was used to detect the distribution of serpins in the A-MSG at the fifth day of the fifth instar. Strong signals could be observed in the lumen, and weak signals could be detected in the gland cells (Fig. 4E), suggesting that serpins were secreted from the A-MSG cells into the lumen.

### Identification of the physiological substrates of serpin18 in the silk gland

The expression patterns and localization of serpin18 and its paraologs suggested that the lumen of the MSG is the possible site where serpin18 to exerts the cysteine protease inhibitory activity. Meanwhile, we identified a cathepsin L-like cysteine protease, fibroinase, in the content of silk gland by LC-MS/MS (unpublished data). Fibroinase activity has also been detected in the silk gland in the fifth instar larva^25^, and previous immunofluorescence analysis showed that fibroinase could be observed in the lumen contents of the silk gland in the anterior part of the MSG in the fifth instar larva^26^. The concurrence of the temporal–spatial distribution between serpins and fibroinase suggests that fibroinase might be a physiological target of serpin18.

**Figure 5:**
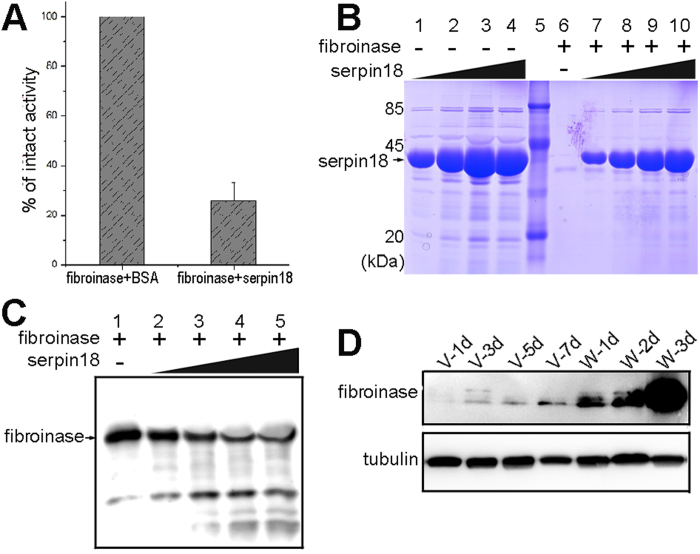
Inhibitory activity of serpin18 towards fibroinase. (**A**) The bar graph diagrams of residual activities of fibroinase incubated with serpin18 at molar ratios of 5. (**B**) The reaction between serpin18 and fibroinase were analyzed by SDS-PAGE method followed by Coomassie-staining and Lanes 1 to 4: 0.5 mg/ml, 1.0 mg/ml, 1.5 mg/ml, 2.0 mg/ml serpin18 without fibroinase; Lane 5, protein marker; Lane 6, 0.25 mg/ml fibroinase; Lanes 7 to 10: samples corresponding to lanes 1 to 4, respectively, with 0.25 mg/ml fibroinase. (**C**) Western blot analysis the reaction between serpin18 and fibroinase using the antibody of fibroinase. Lane 1: 0.25 mg/ml fibroinase; Lanes 2 to 5: 0.5 mg/ml, 1.0 mg/ml, 1.5 mg/ml, 2.0 mg/ml serpin18 with 0.25 mg/ml fibroinase. (**D**) Western blot analysis for fibroinase in the developmental stages of the fifth instar and the wandering periods in the A-MSG. The gels were run under the same experimental conditions and the full length blots and gels are presented in Supplementary Figure S7.

To determine whether serpin18 could inactivate fibroinase, we purified the naturally occurring fibroinase protein from silk gland lysates as previously described^27^,^28^ and tested the inhibiton of fibroinase by serpin18. Serpin18 showed significant inhibitory activity against fibroinase, with its proteolytic activity showing a 73% reduction (Fig. 5A). Similar to the inhibition of papain by serpin18, the reduction in fibroinase was not a consequence of the formation of a stable serpin-fibroinase complex, but instead the digestion of both protease and serpin was observed (Fig. 5B,C).These results suggested that fibroinase is one of the physiological targets of serpin18. Notably, fibroinase from *B. mori* silk glands is a cathepsin L-like cysteine protease that catalyzes the hydrolysis of liquid silk proteins (including fibroin and sericin)^28^,^29^. Thus, we conclude that serpin18 plays a physiological role in regulating the activity of fibroinase and protecting silk proteins from degradation during *B. mori* development.

## Discussion

The X-ray structure of serpin18 from *B. mori* shows a typical serpin fold, but a different RCL structure. The RCL of serpin18 includes only 13 amino acids, whereas the RCL of classical serpins usually contain about 20 residues (Fig. 2D). The difference in the RCL between serpin18 and classical serpins is mainly reflected in the length of the N-terminal portion of the RCL up to the scissile bond. In previous studies of serpins, the length is almost always 17 residues (P17–P1), with the exceptions of the cowpox virus crmA^30^, human C1 inhibitor^31^ and human α2-antiplasmin^32^, which are each 16 residues long. In serpin18, the length of the N-terminal portion of the RCL up to the scissile bond is only 11 amino acids. These differences in the length of the RCL between serpin18 and its counterparts imply that serpin18 might have a distinct inhibition model, because the invariance of RCL length is directly related to the inhibition mechanism^23^,^33^. Our studies found that the inhibition of papain is not a consequence of the formation of a stable complex with serpin18, but instead occurs because of the autolytic digestion of papain into small fragments, presumably by an active papain (Fig. 4C–E). In the inhibition of papain by typical serpin, human α-antithrombin, two proteins form a thioacyl-intermediate complex, and then the papain trapped in the complex is rapidly digested into smaller peptides^34^. Previous studies have shown that those target proteases are more susceptible to proteolysis in a complex with inhibitors compared to the free protein^16^,^35^. These data indicate that the degradation of papain could also be a consequence of the formation of a thioacyl-intermediate complex with serpin18, and the complex-bound papain has been subjected to conformational distortion and degradation. Moreover, in the reaction of papain and α-antithrombin, the inhibitor is converted into a reactive bond-cleaved form, which tend to form stable dimer with an intact inhibitor^34^. By contrast, in the reaction of cysteine protease and serpin18, the inhibitor is degraded as a substrate. The different fates of the two serpins in the reaction with papain might reflect the length of the N-terminal portion of RCL, which is significantly shorter in serpin18 than that in α-antithrombin. Zhou *et al*. examined the effects of various loop lengths within α1-antitrypsin on the inhibition of factor Xa and thrombin^33^. The shortening of one or two residues of the RCL reduces the efficiency of inhibition and increases the stability of the covalent complex. Notably, the deletion of more than two residues completely converted the serpin from an inhibitor into a substrate, because the RCL became too short to recruit the protease to the bottom of serpin^33^. In serpin18 studied here, the length of the N-terminal portion of RCL up to the scissile bond is only 11 amino acids, which is equivalent to shortening six amino acids from the RCL of typical serpins. It is therefore unsurprising that serpin18 was also digested both in the inhibition of papain and fibroinase. Besides the length of RCL, the composition of RCL is also critical to the inhibitory mechanism. In the classical serpin, the insertion of reactive center loop involves incorporation of a previously exposed loop region into the interior of the protein, and the P14 residue is the first side chain to become buried^23^. The classical serpins commonly have a noncharged residues (usually the Thr) at P14 position, in contrast to the serpin18 that has a Glu (Glu355) at the critical position. Since the P14 side chain would normally become buried upon reactive center loop insertion, the Glu355 would be much less favorable and probably increase the activation energy for insertion. In addition, the residues P16 Pro, P15 Thr and P12 Gln in serpin18 might be also preclude the insertion of the reactive loop in to the β-sheetA, because the bigger the side chain that needs to be inserted, the biggerthe cavity that need to be created in the interior of serpin body that to accommodate it. It is therefore unsurprising that the mutation of P14 (E355A) in serpin18 did not prompt the insertion of reactive center loop (Fig. S8).

**Figure 6:**
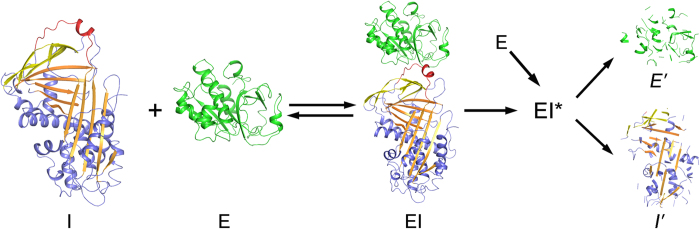
Mode of the reaction of serpin18 with cysteine protease. I, serpin18 (PDB code: 4R9I); E, cysteine protease (papain, PDB code: 1PPP); EI, Michaelis-like complex (constructed by docking the X-ray structures of serpin18 and papain); EI*, thioacyl-intermediate; *E′*, degraded protease and *I′*, degraded serpin18.

Based on our structural observations and previous studies, we proposed a unique inhibitory model for serpin (Fig. 6). First, similar to the inhibition of trypsin by *Manduca* serpin 1B^36^, serpin18 interacts with the protease, and forms a initial noncovalent Michaelis-like complex that involves slight conformational changes within the body of protease and serpin18. Second, the active site of the protease then attacks the scissile bond V362–A363 in the RCL of serpin18. This reaction proceeds in a manner similar to that between serpins and serine protease, from an initial Michaelis complex to the acyl-intermediate complex, in which both the serpin and protease undergo conformational changes to accommodate the transformation. However, during the formation of the thioacyl-intermediate, the RCL of serpin18 cannot become inserted into the β-sheet A and drag the enzyme to the bottom of serpin18. Third, the active site of cysteine protease becomes structurally loosed, as a consequence of a conformational change, becomes susceptible to proteolysis and is then cleaved by other active enzymes. Meanwhile, the complex-bound serpin18 is degraded as a normal substrate. Finally, the thioester bond in the thioacyl-intermediate is hydrolyzed, resulting in the degradation of serpin18 and cysteine protease.

As an inhibitor of cysteine protease, serpin18 is mainly located in the anterior region of the MSG lumen at the fifth instar larval stage. The fibroinase is also observed in the lumen contents of A-MSG in fifth instar larva^26^. Additionally, serpin18 shows robust inhibitory activity towards fibroinase. Thus, we conclude that fibroinase could be a major physiological target of serpin18, but we do not exclude the possibility that serpin18 may have additional targets. The expression pattern analysis shows that fibroinase is weakly observed in the fifth instar stage and mainly observed in the spinning period in the A-MSG (Fig. 5D), whereas the three serpins disappeared at this stage (Fig. 4C). A relatively high fibroinase activity in the larva stage was also observed in the wandering period^25^. Therefore, we speculate that active proteases in silk glands are restricted by serpins until the wandering stage. As serpins quickly disappear at the wandering stage, proteases in the silk gland begin to show bioactivities. In addition, fibroinases were thought to have dual physiological roles in the silk gland during different developmental stage of *B. mori*. First, they promote the digestion of obsolete proteins and cellular organelles in lysosomes. Second, they digest silk proteins in the lumen of silk gland after spinning^25^. To avoid the degradation of accumulated silk proteins in the silk gland lumen at the fifth instar larva, abundant serpins are synthesized and secreted into the lumen to regulate the activity of fibroinases before wandering. However, extra silk proteins need to be cleaned up after spinning, because extra proteins would infect the apoptosis of the silk gland at the pupal stage. Thus, serpin expression has evolved to be down-regulated in the silk gland lumen before apoptosis, and thereafter the proteases begin to play the dominant role.

## Methods

### Construction, expression, and purification of serpin18

The open reading frame of *SERPIN18* without the signal peptide was amplified by PCR, using *B. mori* (strain p50, Dazao) silk gland cDNA as the template, and cloned into a pET28a-derived vector. This construct with an N-terminal hexahistidine-tag was transformed into *E. coli* BL21 (DE3) (Novagen, Madison, WI) and induced with 0.2 mM isopropyl-β-D-thiogalactoside (IPTG) at 16 °C for 20 h when OD_600nm_ reached 0.6. Cells were harvested by centrifugation at 8000 *g* for 10 min and resuspended in lysis buffer (20 mM Tris-HCl, pH 6.8, 200 mM NaCl). After 5 min of sonication and centrifugation at 12,000 *g* for 25 min, the supernatant containing the soluble target protein was loaded onto a HiTrap nickel-chelating column (GE Healthcare) that had been equilibrated with binding buffer (20 mM Tris-HCl, pH 6.8, 200 mM NaCl). The target protein was eluted with 250 mM imidazole buffer and further loaded onto a HiLoad 16/60 Superdex 200 column (GE Healthcare) equilibrated with 20 mM Tris-HCl, pH 6.8, 50 mM NaCl. Fractions containing the target protein were pooled and concentrated to 20 mg/mL. The purity of protein was estimated on SDS-PAGE and the protein sample was stored at −80 °C. The mutant proteins were expressed, purified, and stored using the same protocol as the wild-type protein.

Selenomethionine (SeMet)-labeled serpin18 proteins were expressed in *E. coli* strain B834 (DE3) (Novagen). A culture of transformed cells was inoculated into LB medium and incubated at 37 °C overnight. The cells were cultured in SeMet medium (M9 medium with 25 mg/L SeMet and other essential amino acids at 50 mg/L) to an OD_600nm_ of 0.6–0.8. Protein expression and purification were the same as those for the native protein.

### Crystallization and data collection

Both native and SeMet substituted crystals of serpin18 were obtained at 289 K using the hanging drop vapor-diffusion techniques, with the initial condition by mixing 1 μL of 20 mg/mL protein sample with equal volume of mother liquor 1.5 M sodium citrate, pH 6.5. The native crystals grew to approximately 0.5 × 0.5 × 0.3 mm in about 3 days. SeMet derivative crystals were grown under the same conditions. The crystals were soaked into the cryoprotectant of reservoir solution supplemented with 25% glycerol and flash-cooled at 100 K in liquid nitrogen. Both the native and SeMet derivative data for single crystals were collected at radiation wavelength of 0.9791 Å at Shanghai Synchrotron Radiation Facility, Shanghai Institute of Applied Physics, Chinese Academy of Sciences, using beamline BL17U at 100 K with a Q315r CCD (MARresearch). Data processing and scaling were performed using the HKL3000 package^37^.

### Structure determination and refinement

The crystal structure of serpin18 was determined by the single-wavelength anomalous dispersion phasing method from a single SeMet-substituted protein crystal to a maximum resolution of 1.60 Å. The *AUTOSO*L program from *PHENIX*^38^ was used to locate the heavy atoms, and the phase was calculated and further improved with the program *OASIS*^39^. Electron density maps showed clear features of secondary structural elements. Automatic model building was carried out using *AutoBuild* in *PHENIX*. Afterwards, the initial model was used for the molecular replacement process against the native dataset at 1.65 Å with *MOLREP*^40^. Refinement was carried out using *REFMAC*^41^ and *Coot*^42^. The overall assessment of model quality was performed using *MolProbity*^43^. The crystallographic parameters of the structures are listed in Table 1. All structural figures were prepared with *PyMOL*.

### Inhibitory activity assay

To detect the activity of inhibitors, the proteases were preincubated with serpin18 (at 1:5 molar ratios) for 30 min at 37 °C. Trypsin (SIGMA T1426), chymotrypsin (SIGMA C4129), elastase (SIGMA 45124), subtilisin (SIGMA P5380), protease K (ROCHE 11060325), papain (SANGON P16J12) and cathepsin L (SIGMA SRP0291) was respectively used as target proteases. Then, the residual protease activity was assayed by using FITC-Casein protease substrates (GBios). A 200 μL reaction containing appropriate proteases, 5 μg substrates, and 100 mM Tris-HCl buffer containg 20 mM calcium chloride (pH 7.4), was incubated for 1 h at 37 °C. Substrate hydrolysis was monitored by measuring excitation and emission wavelength at 485 nm and 528 nm in a Fluorescence Microplate Reader (BioTek). The percentage of inhibition was assessed by the following formula: % inhibition = (1–inhibited rate/uninhibited rate) ×100.

### Samples preparation and expression analysis of serpins

*B. mori* larvae of Dazao strain (maintained in the State Key Laboratory of Silkworm Genome Biology at Southwest University of China) were reared at 25 °C under a photoperiod of 12 h of light and 12 h of dark for the experiments. Silkworm larvae samples from the fifth instar were prepared for RNA isolation or protein extraction. RNAs were extracted using TRIZOL reagent (Invitrogen). Contaminating genomic DNA was digested using RNasefree DNase I (Promega) for 30 min at 37 °C. cDNAs were obtained using M-MLV reverse transcriptase (Invitrogen) at 42 °C. All cDNA samples were normalized using *B. mori* actin A3 as an internal control (forward primer: 5′-AAC ACC CCG TCC TGC TCA CTG-3′; reverse primer: 5′-GGG CGA GAC GTG TGA TTT CCT-3'). Gene specific primers were designed and presented in Table S2. PCR amplification was performed in a total reaction volume of 25 μL containing normalized cDNA, PCR conditions were as follows: 94 °C for 4 min followed by 30 cycles of 94 °C for 40 s; 56 °C for 40 s; and 72 °C for 55 s. PCR products were analyzed on a 1.5% agarose gel.

Protein samples were extracted from *B. mori* silk gland in 100 mM Tris-HCl buffer (pH 7.4) by homogenized using liquid nitrogen. All the protein samples were centrifuged at 16,000 *g* for 20 min at 4 °C, and the supernatants were collected. The extracted proteins were separated by SDS-PAGE and identified by western blot analysis and MALDI-TOF Mass spectrometry.

## Additional Information

**How to cite this article**: Guo, P.-C. *et al*. Structural insights into the unique inhibitory mechanism of the silkworm protease inhibitor serpin18. *Sci. Rep*. **5**, 11863; doi: 10.1038/srep11863 (2015).

## Supplementary Material

Supplementary Information

## Figures and Tables

**Table 1:** Data collection and refinement statistics.

	SeMet-serpin18	serpin18
Data collection		
Space group	*P*4_1_2_1_2	*P*4_1_2_1_2
Unit cell (Å,°)	109.64, 109.64, 72.92 90, 90, 90	109.30, 109.30, 72.55 90, 90, 90
Molecules per asymmetric unit	1	1
Resolution range (Å)^a^	50.00–1.60 (1.63–1.60)^a^	50.00–1.65 (1.71–1.65)^a^
Unique reflections	58,521 (2,856)^a^	53,389 (5, 240)^a^
Completeness (%)	100.0 (100.0)^a^	99.9 (100.0)^a^
<I/σ(I)>	83.52 (13.46)^a^	23.74 (7.44)^a^
R_merge_^b^ (%)	19.4 (69.7)^a^	10.1 (44.5)^a^
Average redundancy	29.0	12.1
Structure refinement		
Resolution range (Å)		50.00–1.65 (1.69–1.65)^a^
R-factor^c^/R-free^d^ (%)		17.7/20.7
Number of protein atoms		3032
Number of water atoms		518
RMSD^e^ bond lengths (Å)		0.011
RMSD bond angles (º)		1.496
Mean B factors (Å^2^)		21.07
Ramachandran plot^f^		
Most favored (%)		98.4
Additional allowed (%)		1.6
PDB entry		4R9I

^a^Values in parentheses are for the highest resolution shell.

^b^R_merge_ = ∑_hkl_∑_i_|I_i_(hkl)–<I(hkl)>| / ∑_hkl_∑_i_|I_i_(hkl)|, where I_i_(hkl) is the intensity of an observation and <I(hkl)> is the mean value for its unique reflection; Summations are over all reflections.

^c^R-factor = ∑_h_||Fo(h)|–|Fc(h)||/∑_h_|Fo(h)|, where Fo and Fc are the observed and calculated structure-factor amplitudes, respectively.

^d^R-free was calculated with 5% of the data excluded from the refinement.

^e^Root-mean square-deviation from ideal values.

^f^Categories as defined by *MolProbity*.
