# Supplementary material for: Structural insights into the unique inhibitory mechanism of the silkworm protease inhibitor serpin18
Source: Sci Rep. 2015 Jul 7;5:11863. doi: 10.1038/srep11863 (PMC4493575; doi:10.1038/srep11863)
Supplement: Supplementary Information [file srep11863-s1.pdf]

Supplementary Information

Structural insights into the unique inhibitory mechanism of the silkworm protease inhibitor serpin18

Peng-Chao Guo[§], Zhaoming Dong[§], Ping Zhao, Yan Zhang, Huawei He, Xiang Tan, Weiwei Zhang and Qingyou Xia*

State Key Laboratory of Silkworm Genome Biology, Southwest University, 216, Tiansheng Road, Beibei, Chongqing 400716, People's Republic of China.

Specificity detection of anti-serpin16 polyclonal antibody

To explore the three serpins (serpin16, 18 and 22) expression pattern at the protein level, we produced the anti-serpin16 polyclonal antibody and determined its specificity by a combination of two-dimensional gel electrophoresis (2-DE) and western blot. Two spots were observed in the western blot after reaction with the anti-serpin16 polyclonal antibody (Fig. S5A and S5B). The spot 1 was identified as serpin16 and the spot 2 was serpin18 and 22 by MALDI-TOF MS analysis (Fig. S5C, S5D and Table S1). Bioinformatic analysis showed that the molecular weight and isoelectric point of serpin 16 (44.36/4.50) are higher than that of serpin18 (44.01/4.24, Table S1A), which is consistent with the 2-DE result. These results suggested that the polyclonal antibody of serpin16 had cross reaction with serpin18 due to their amino acid sequences share high degree of homology. Therefore, this antibody could be used to investigate the expression and localization of the three serpins.

Figure S1.

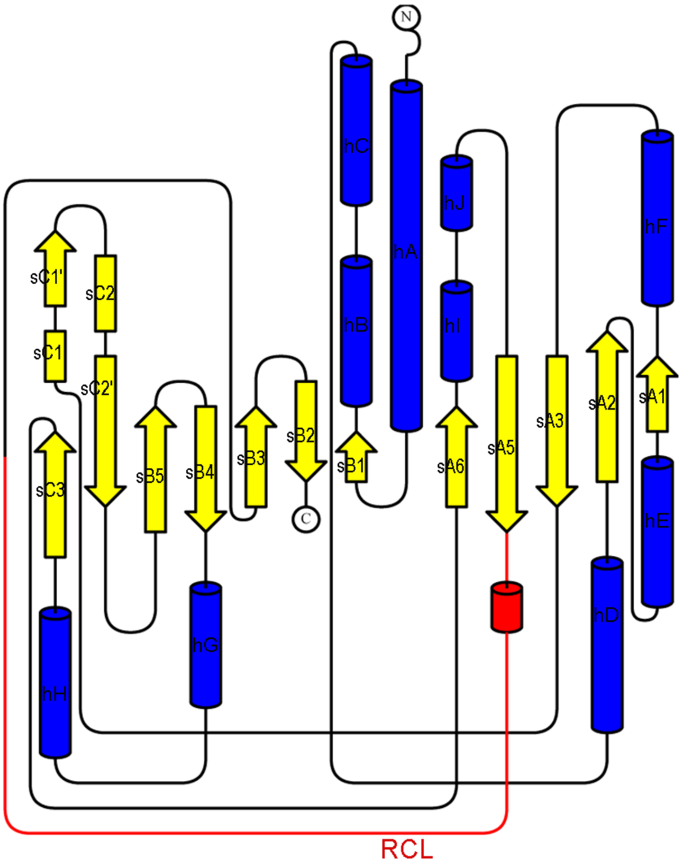

Figure S1. Topology diagram of serpin18. Related to Figure 1.

Figure S2.

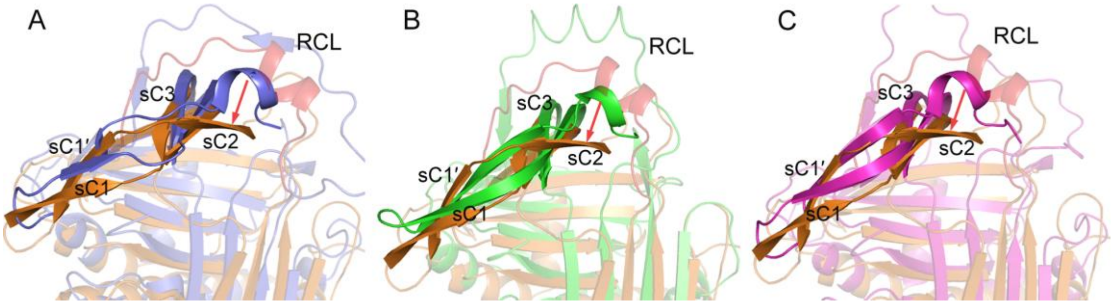

Figure S2. Close-up view of β-sheet C. Related to the Figure 2.

Comparision of the structure of β-sheet C between serpin18 (orange) and (**A**) human plasminogen activator inhibitor-1 (PDB: 1DB2, blue), (**B**) human plasminogen activator inhibitor-1 complexed with plasminogen activator (PDB: 3PB1, lightmagenta) and (**C**) Ovalbumin (PDB: 1OVA, green). All figures were prepared using *PyMOL*.

Figure S3.

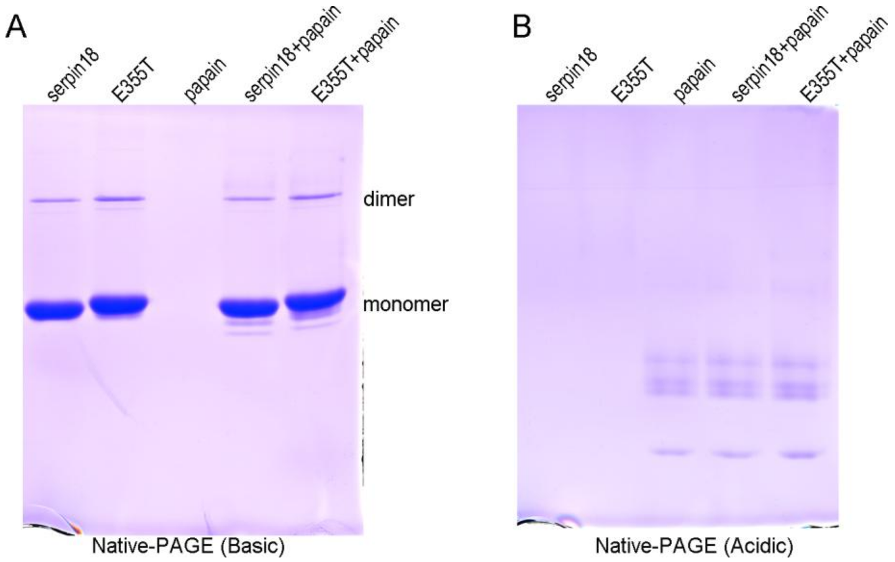

Figure S3. Native-PAGE analysis of the reaction between serpin18 (including the wild-type and the mutant E355T) and papain were analysed by Coomassie brilliant blue staining under the non-denaturing conditions. Related to Figure 3.

Figure S4.

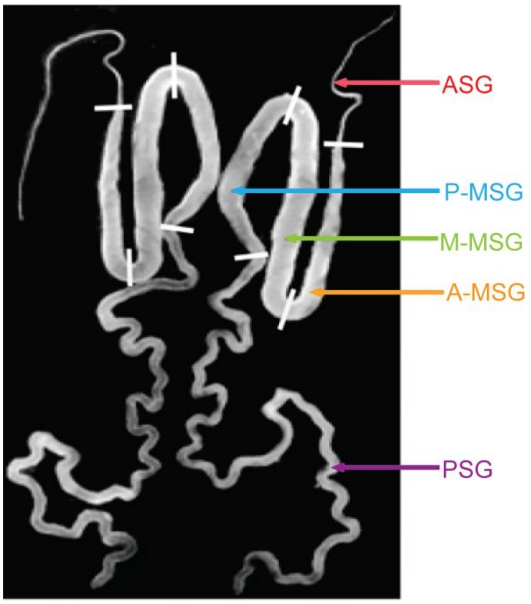

Figure S4. Larval silk gland of *B. mori*. Related to Figure 4.

Abbreviations are as follows: ASG, anterior silk gland; PSG, posterior silk gland; MSG, middle silk gland. Three subsections of MSG (anterior/middle/posterior region

of the middle silk gland, A/M/P-MSG) are prepared according to the morphology.

Figure S5.

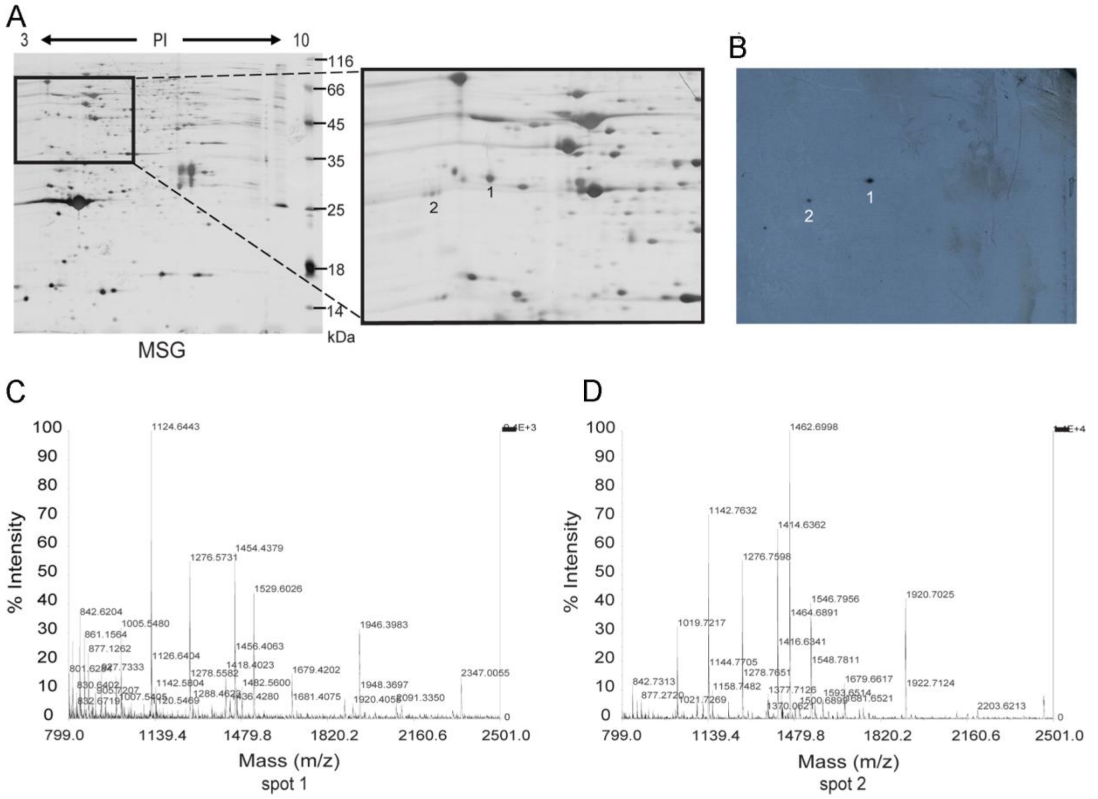

Figure S5. Western blot analysis for serpins in a 2-DE gel from MSG. Related to supplemental data.

(**A**) Silver-stained 2-DE graph; (**B**) Immunological detection of *B. mori* serpins with anti-serpin 16 polyclonal antibody. MALDI-TOF MS spectra of spot 1 (**C**) and spot 2 (**D**) are shown following trypsin digestion. The detailed information of identified result of spot1 and 2 was shown in Table S1.

Figure S6.

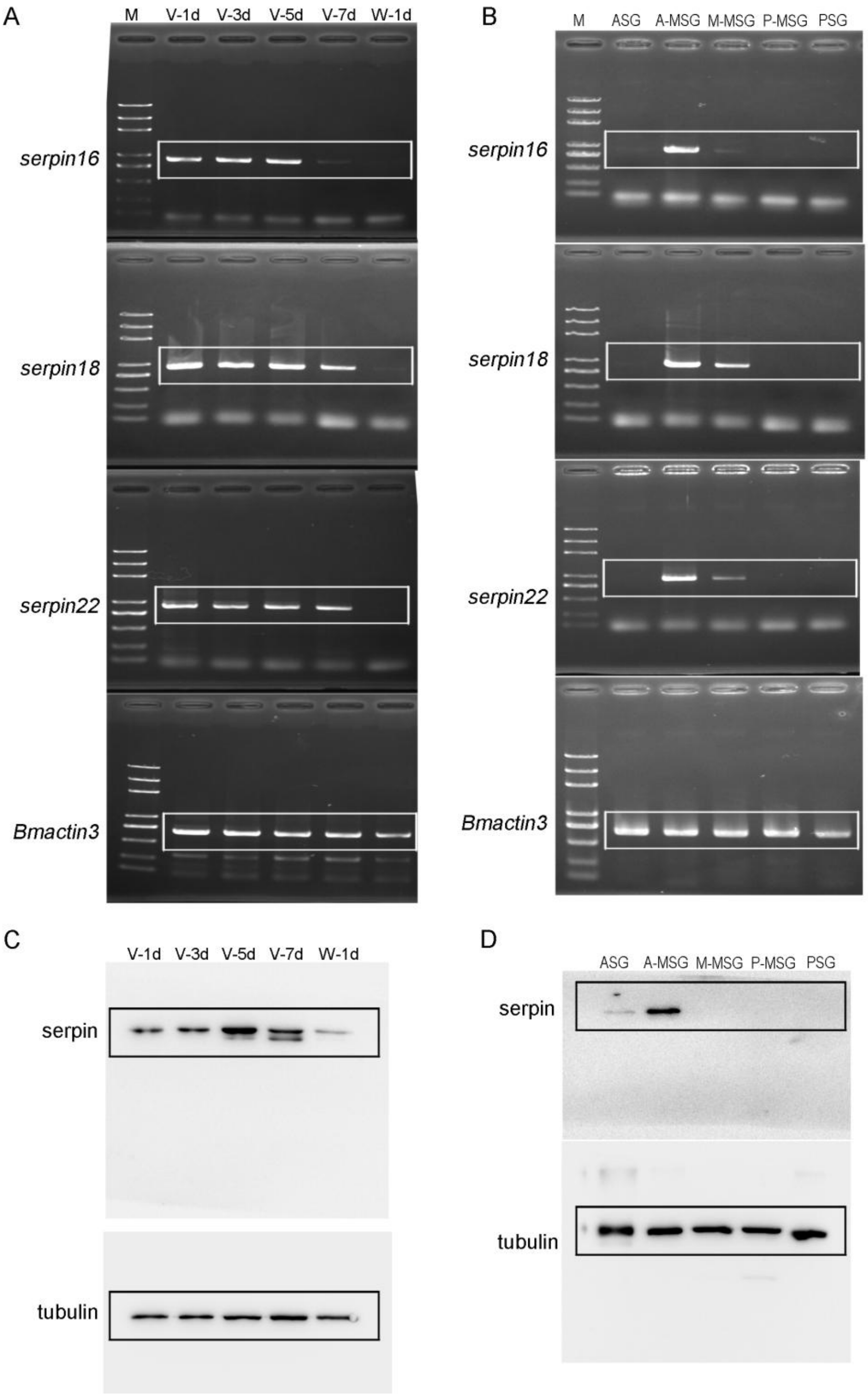

Figure S6. Expression patterns of serpins in the silk gland. Related to Figure 4.

(**A**) Expression patterns of *serpins* genes in the day1, 3, 5, 7 of the fifth instar and the hour 12 after wandering. (**B**) Expression patterns of serpin in the different segments of the silk gland in the day 5 of the fifth instar. Western blot analysis for serpin18 and its paralogs in the (**C**) developmental stages of the fifth instar and (**D**) different segments

of the silk gland.

Figure S7.

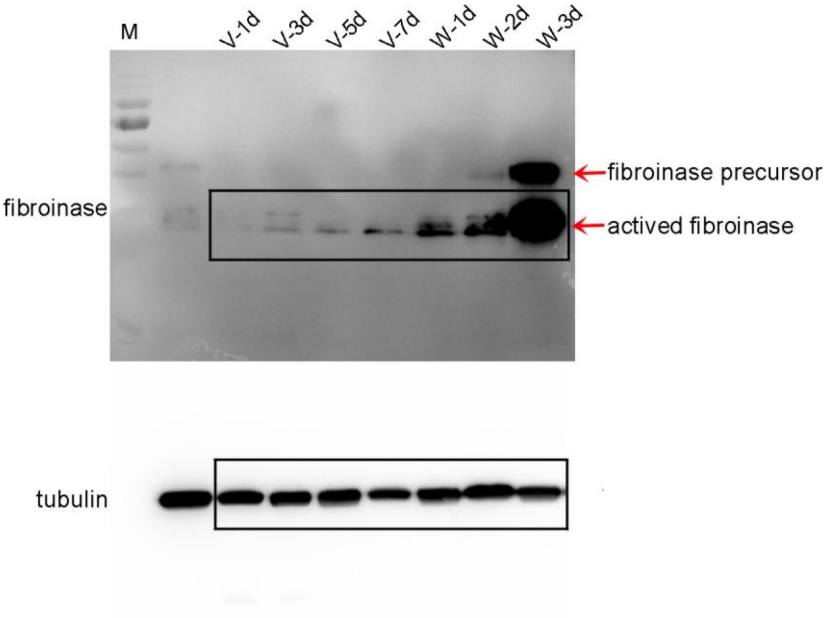

Figure S7. Western blot analysis for fibroinase in the developmental stages of the fifth instar and the wandering periods in the A-MSG. Related to Figure 5D.

Figure S8.

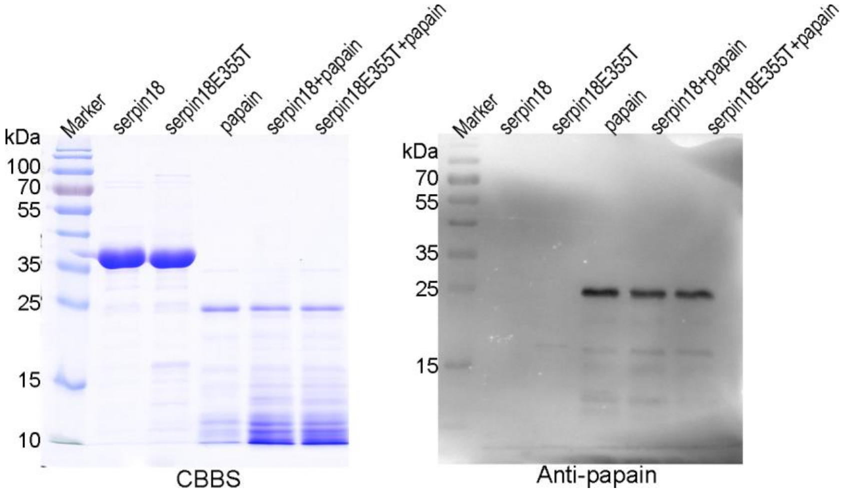

Figure S8. SDS-PAGE of the reaction between the mutant serpin18E355T and papain were analysed by Coomassie brilliant blue staining (CBBS) and western blot. Related to the Figure 2C.

Table S1. The match result of spot1 and 2 identified by GPMAW

A. Information of identified proteins

Spot No.	Protein name	Unique peptide Number	Peptide Number	NCBI entry GI	MW(kDa)	PI
spot 1	serpin16	14	19	gi|226342892	44.363	4.50
spot 2	serpin18	5	16	gi|226342898	44.011	4.24
spot 2	serpin22	1	11	gi|226342906	44.024	4.25

B. Detailed information of spot 1

Measured	Computed	Overlap	Corresponding sequence	Corresponding protein
897.672	896.979	A/0	R FCEANTR L	serpin16
986.797	986.181	A/0	K IFQFYLR G	serpin16
1005.786	1005.138	A/1	R DYVPEVKR A	serpin16
1096.736	1096.164	A/0	K QAWYSEGAGK Y	serpin16, serpin18, serpin22
1120.823	1120.31	A/0	R AGLLTELFEK N	serpin16, serpin18, serpin22
1124.907	1124.389	A/0	K LAVPIELTLR D	serpin16
1149.813	1149.309	A/0	K YAEIENLGIK I	serpin16
1166.754	1166.326	A/1	R LRFCEANTR L	serpin16
1276.863	1276.497	A/1	K RAGLLTELFEK N	serpin16, serpin18, serpin22
1418.729	1418.663	A/0	K SGYISHMLSHMR I	serpin16
1434.72	1434.481	A/0	K NFDGFDTVYDNK S	serpin16, serpin18, serpin22
1482.931	1482.743	A/1	K LAVPIELTLRDSR D	serpin16
1524.742	1524.674	A/0	K NIDYCNDAVATIR D	serpin16
1528.949	1528.898	A/1	K YLKLAVPIELTLR D	serpin16
1679.811	1679.801	A/0	R SEDEVVGVNQIAYEK T	serpin16
1679.811	1679.847	A/1	K EDTTAELYKNLNLR S	serpin16, serpin18
1945.889	1946.231	A/1	K LRDPATLPYILTQTESK Y	serpin16
2010.686	2011.244	A/0	R LCGIGIDQWFQYEPER N	serpin16
2289.633	2290.515	A/1	K NLNLRSEDEVVGVNQIAYEK T	serpin16

C. Detailed information of spot 2

Measured	Computed	Overlap	Corresponding sequence	Corresponding protein
1019.72	1019.165	A/1	R DYIPEVKR A	serpin18
1096.649	1096.164	A/0	K QAWYSEGAGK Y	serpin16, serpin18, serpin22
1120.740	1120.273	A/1	K DLSGAFNKLR D	serpin22
1120.744	1120.31	A/0	R AGLLTELFEK N	serpin16, serpin18, serpin22
1142.763	1142.428	A/0	K LAVPIELTMR D	serpin18
1149.833	1149.309	A/0	K YAEIENLGIK V	serpin18, serpin22
1221.685	1221.332	A/1	R DSRDYIPEVK R	serpin18
1276.76	1276.497	A/1	K RAGLLTELFEK N	serpin16, serpin18, serpin22
1414.637	1414.604	A/0	K SGYISYMLSHTR L	serpin18, serpin22
1434.575	1434.481	A/0	K NFDGFDTVYDNK S	serpin16, serpin18, serpin22
1452.663	1451.555	A/0	K NIDYSNDAVATIR D	serpin18, serpin22

1500.711	1500.782	A/1	K LAVPIELTMRDSR D	serpin18
1546.796	1546.937	A/1	K YLKLAVPIELTMR D	serpin18
1650.716	1650.846	A/0	R DPATLAYILTQTESK Y	serpin18, serpin22
1679.665	1679.847	A/1	K EDTTAELYKNLNLR S	serpin16, serpin18
1737.617	1737.883	A/0	K QIDISFTALDGTQSNK Q	serpin16, serpin18, serpin22
1919.73	1920.193	A/1	K LRDPATLAYILTQTESK Y	serpin18, serpin22

Table S2. List of primers for semi-quantitative RT-PCR

Primer	Sequence (5´ to 3´)	length(bp)
serpin16-F	GGCGTCAACCAGATTGCCT	897
serpin16-R	TCAGTGCGTGATAAGGCCAACG	
serpin18-F	AACGTTAACCAGGCCGTCA	897
serpin18-R	TCAGTTTGTAATAAGGCCAATG	
serpin22-F	TACGTCAACCAGGCCGTCA	897
serpin22-R	TCAGGTCGTAATAATGCCAATG	
